# 768 Utilization of a University Physical and Occupational Therapy Pro Bono Clinic for a Burn Survivor

**DOI:** 10.1093/jbcr/irae036.310

**Published:** 2024-04-17

**Authors:** Elizabeth Richardson, Debbie Prouty, Anju Saraswat, John Kevin Bailey, James H H Holmes

**Affiliations:** Atrium Health Wake Forest Baptist, Winston-Salem, NC; Wingate University, Wingate, NC; Wake Forest University School of Medicine, Winston-Salem, NC; Atrium Health Wake Forest Baptist, Winston-Salem, NC; Wingate University, Wingate, NC; Wake Forest University School of Medicine, Winston-Salem, NC; Atrium Health Wake Forest Baptist, Winston-Salem, NC; Wingate University, Wingate, NC; Wake Forest University School of Medicine, Winston-Salem, NC; Atrium Health Wake Forest Baptist, Winston-Salem, NC; Wingate University, Wingate, NC; Wake Forest University School of Medicine, Winston-Salem, NC; Atrium Health Wake Forest Baptist, Winston-Salem, NC; Wingate University, Wingate, NC; Wake Forest University School of Medicine, Winston-Salem, NC

## Abstract

**Introduction:**

Burn survivors often have ongoing therapy needs after injury and skin grafting to address the multi-system impact of burns on their functional outcomes. A challenge to follow-up care can be related to social and political determinants of health. These determinants can negatively impact a patient’s recovery including the opportunity to receive medical services after their hospitalization. We sought to bridge this gap by locating and utilizing pro bono physical and occupational therapy services that have not been previously used by our burn center.

**Methods:**

We referred a patient to a local University Doctor of Physical Therapy and Occupational Therapy pro bono clinic who is undocumented, uninsured and has limited English-speaking proficiency. The patient sustained 58% total body surface area full-thickness flame burns involving the head, face, neck, chest, abdomen, back, buttocks, bilateral lower extremities, bilateral upper extremities, and hands after a motor vehicle collision. He underwent seven excision and skin grafting interventions over his 89-day hospital admission. His inpatient rehabilitation stay was 20 days. We collected data from the Doctor of Physical Therapy and Occupational Therapy students who treat this patient to understand their experience. We also had dialogue with the patient on his return to the burn clinic and with one of the physical therapy professors for anecdotal perspectives of the experience. It is noted that the professors are actively involved in the treatment of the patient in the clinic.

**Results:**

We collected 10 student surveys at a 76% completion rate. 75% of the students state knowledge of treatment strategies are the most challenging aspect of treating a burn survivor. This is followed by 12.50% stating pain management is most challenging. 55.56% of the students report limited confidence, 33.33% are not confident and 11.11% are fully confident with treating burn survivors. 70% of the students state treating a burn survivor during school impacts decision-making for their future career path. 90% report a pro bono clinic is a positive resource for the burn survivor population while 10% are undecided. Within the open-ended questions students request more robust education as well as guidance from the referring provider for treating patients referred to the pro bono clinic.

**Conclusions:**

Doctor of Physical and Occupational Therapy program pro bono clinics have the opportunity to fill two needs in the burn community. One is the exposure of students to burn survivors to gain experience in treatment implementation following their didactic burn lecture. The second is to address long-term therapy needs of burn survivors in the setting of limited resources.

**Applicability of Research to Practice:**

These survey results demonstrate there is potential for building relationships with pro bono clinics connected to universities. However, there is a need for increased communication and education to improve the patient and student experience.

